# Examining *Plasmodium falciparum* and *P. vivax* clearance subsequent to antimalarial drug treatment in the Myanmar-China border area based on quantitative real-time polymerase chain reaction

**DOI:** 10.1186/s12879-016-1482-6

**Published:** 2016-04-16

**Authors:** Eugenia Lo, Jennifer Nguyen, Winny Oo, Elizabeth Hemming-Schroeder, Guofa Zhou, Zhaoqing Yang, Liwang Cui, Guiyun Yan

**Affiliations:** Program in Public Health, University of California at Irvine, Irvine, CA 92697-4050 USA; Department of Pathogen Biology and Immunology, Kunming Medical University, Kunming, China; Department of Entomology, Pennsylvania State University, University Park, PA USA

**Keywords:** *P. falciparum*, *P. vivax*, Malaria, Artemisinin-combined therapy, Quantitative PCR, Parasite clearance, Resistance genes, Microsatellite

## Abstract

**Background:**

Recent emergence of artemisinin-resistant *P. falciparum* has posed a serious hindrance to the elimination of malaria in the Greater Mekong Subregion. Parasite clearance time, a measure of change in peripheral parasitaemia in a sequence of samples taken after treatment, can be used to reflect the susceptibility of parasites or the efficiency of antimalarials. The association of genetic polymorphisms and artemisinin resistance has been documented. This study aims to examine clearance time of *P. falciparum* and *P. vivax* parasitemia as well as putative gene mutations associated with residual or recurred parasitemia in Myanmar.

**Methods:**

A total of 63 *P. falciparum* and 130 *P. vivax* samples collected from two internally-displaced populations and one surrounding village were examined for parasitemia changes. At least four samples were taken from each patient, at the first day of diagnosis up to 3 months following the initial treatment. The amount of parasite gene copy number was estimated using quantitative real-time PCR based on a species-specific region of the 18S rRNA gene. For samples that showed residual or recurred parasitemia after treatment, microsatellites were used to identify the ‘post-treatment’ parasite genotype and compared such with the ‘pre-treatment’ genotype. Mutations in genes *pfcrt*, *pfmdr*1, *pfatp*6, *pfmrp*1 and *pfK*13 that are potentially associated with ACT resistance were examined to identify if mutation is a factor for residual or persistent parasitemia.

**Results:**

Over 30 % of the *P. falciprium* infections showed delayed clearance of parasitemia after 2–3 days of treatment and 9.5 % showed recurred parasitemia. Mutations in codon 876 of the *pfmrp1* corroborated significance association with slow clearance time. However, no association was observed in the variation in *pfmdr1* gene copy number as well as mutations of various codonsin*pfatp6, pfcrt,* and *pfK13* with clearance time. For *P. vivax*, over 95 % of the infections indicated cleared parasitemia at days 2–3 of treatment. Four samples were found to be re-infected with new parasite strains based on microsatellite genotypes after initial treatment.

**Conclusion:**

The appearance of *P.falciparum* infected samples showing delayed clearance or recurred parasitemia after treatment raises concerns on current treatment and ACT drug resistance.

**Electronic supplementary material:**

The online version of this article (doi:10.1186/s12879-016-1482-6) contains supplementary material, which is available to authorized users.

## Background

Internal conflict and associated large-scale human movement in Myanmar during the past few years have in part attributed to an increase of malaria cases within the country and to its spread beyond the national border into Thailand and China [1]. Along with the high malaria burden, multidrug-resistant *Plasmodium falciparum* malaria has also emerged and widespread in endemic areas of the Greater Mekong Subregion (GMS) [1]. In the 1960s and 1970s, chloroquine (CQ) resistance had spread throughout the region and subsequently, in the 1980s, resistance to sulphadoxine and pyrimethamine (SP) was reported [2]. Nevertheless, SP combination is still the drug treatment recommended by WHO for intermittent preventive treatment (IPT) in vulnerable populations because of its safety in pregnant women and infants and its long-lasting action. Following the decline in clinical efficacy of CQ and SP, the artemisinin-based combination therapy (ACT) using the artesunate-mefloquine combination was introduced as first-line treatment in the 1990s [3]. However, the recent emergence of artemisinin-resistant *P. falciparum* in the GMS has posed a serious hindrance to the elimination of malaria [4]. The reduced susceptibility to ACT may have also spread to the African continent where some of the affected countries have adopted ACT as first-line antimalarial treatment [5].

Parasite Clearance Time (PCT), a measure of change in peripheral parasitaemia in a sequence of samples taken after treatment, can be used to reflect the susceptibility of parasites or the efficiency of antimalarials. Typically, malaria parasite densities are expected to be reduced by a factor of 10^8^ after a 3-day treatment course with an ACT, with 95 % of patients’ microscopic results to be negative 48 h after treatment [6]. However, contrary to this expectation, an increasing number of cases of delayed parasite clearance after treatment with an artemisinin derivative have been reported in Cambodia [7–9]. Along the Thailand-Cambodia border, the time to reach the clearance of parasites after artesunate-mefloquine combination therapy has also become longer [10, 11]. In Kenya, over 30 % of children were reported with residual submicroscopic parasitemia after ACT [5]. These children were significantly more likely to experience recurrent parasitemia during follow-up. Parasite clearance time is influenced by parasite drug susceptibility, parasite density before initiation of treatment, and inter-individual differences in antimalarial pharmacokinetics and immunity [12]. A recent study with clonally identical parasites has shown that clearance time was primarily dictated by the parasite’s genetic background and less by host factors, which allows the identification of these parasite factors through genome-wide association [13].

The genetic basis of resistance to antimalarials, such as chloroquine (CQ) and sulfdoxine/pyrimethamine (SP), has been well documented. Numerous molecular studies have indicated multiple independent origins of CQ resistance associated with mutations in the chloroquine-resistance transporter gene (*crt*) [14–16], and the multidrug resistance transporter gene (*mdr*1) [17–19], as well as SP resistance associated with mutations in the genes encoding dihydrofolate reductase (*dhfr*) and dihydropteroate synthase (*dhps*) in *P. falciparum* [20–23]. However, for other antimalarials such as ACT, the molecular mechanism of resistance still remains unclear. Previous studies have shown the association of several mutations with moderately altered susceptibility to one or more artemisinin derivatives. For example, mutations in gene *pfmrp*1 were likely associated with resistance to CQ, mefloquine (MQ), and artemisinin derivatives [24, 25]. Likewise, Gupta et al. [25] indicated signature of positive selection in *pfmrp*1 that was associated with reduced susceptibilities to CQ, MQ, pyronaridine, and lumefantrine in the northeast Myanmar *P. falciparum* isolates. Other studies have shown that changes in amino acids 263 and 769 of the*pfatp*6 gene were related to reduced in vitro artemisinin inhibition [26-28], but no variations were detected in these positions among natural *falciparum* populations [29, 30]. Recently, a strong association was detected between mutations in *pfmdr*1 gene and reduced susceptibility of *P. falciparum* isolates to MQ, artesunate, and quinine in areas along the Thai-Cambodian and Thai-Myanmar borders [18, 30]. Furthermore, several mutations in the*pfK*13-propeller gene (*K*13), PF3D7_1343700, have been reported in the China-Myanmar border area, and those mutations may associate with artemisinin resistance [31].

In Myanmar, reduced susceptibilities to ACT have been continuously reported [32–34]. Detailed monitoring of parasite clearance dynamics after antimalarial treatment is needed to determine whether parasite responsiveness to ACT is changing. For such purposes, quantitative polymerase chain reaction (qPCR) method has been proposed for the analysis of sequentially collected daily filter paper blood samples after initiation of treatment to sensitively detect and quantify parasites below the microscopic threshold [35]. In this study, we aimed to first measure parasitemia level of *Plasmodium falciparum* and *P. vivax* infections detected in Internally Displaced Population (IDP) settlement and surrounding villages of Myanmar over duration of 42 days or longer after initial drug treatment; second, to identity the proportion of individuals with residual/persistent parasitemia or with recurring infections. We then asked whether the recurring infections were attributed to the same or different parasite strain. Third, we compared sequences of a panel of antimalarial drug resistance genes between infections of fast and delayed clearance to examine the associated mutations.

## Methods

### Sample collection

Nearly 100 patients diagnosed with *P. falciparum* and *P. vivax* infections were included in this study. These individuals were selected from clinics/hospitals located in the IDP settlement (Je Yang Hka) and surrounding village (Laiza) in Myanmar from 2011 to 2013. All studied individuals showed fever or malaria-related symptoms at the first day of diagnosis. They were diagnosed with falciparum and/or vivax infection by microscopic examination and later confirmed by PCR assays. Patients diagnosed with uncomplicated *P. falciparum* malaria were treated with dihydroartemisinin-piperaquine (DP) and those with *P. vivax* malaria were treated with chloroquine (CQ). For each patient, at least four samples were taken from day 0 (before antimalarial treatment) and then at days 1, 2, 3, 7, 14, 28, 42, and up to 3 months after beginning of treatment. All samples of each of the patients were run in parallel to estimate the level of parasitemia during treatment. For each sample, 30–50 μl of blood was blotted onto Whatman 3MM filter papers. Filter papers were air-dried and stored in zip-sealed plastic bags with silica gel absorbent at room temperature until DNA extraction. Parasite DNA was extracted from dried blood spots by the Saponin/Chelex method [36]. Samples that showed cleared parasites on day 2 or 3 were classified as fast clearance, whereas those that showed cleared parasites after day 3 of the initial drug treatment were classified as delayed clearance [37–39]. In addition, we calculated parasite reduction ratio after the first 48 h of antimalarial treatment (PRR_48_) as follow: (parasitemia after 48 h of treatment)/(initial parasitemia) [40–42]. We used a PRR value of 0.01 as cut-off, i.e. 99 % of the initial parasitemia cleared after 48 h of drug treatment and compared the results between samples of fast and delayed clearance.

### Quantification of parasitemia by real-time qPCR assays

Quantitative real-time PCR specifically the SYBR Green detection method [43] was employed using *P. falciparum*-specific primers (forward: 5’AGTCATCTTTCGAGGTGACTTTTAGATTGCT-3’; reverse: 5’- GCCGCAAGCTCCACGCCTGGTGGTGC-3’) and *P. vivax*-specific primers (forward: 5’-GAATTTTCTCTTCGGAGTTTATTCTTAGATTGC-3’; reverse: 5’GCCGCAAGCTCCACGCCTGGTGGTGC-3’) that targeted on the plasmodial 18S rRNA region [43]. Amplification was conducted in a 20 μl reaction mixture containing 2 μl of genomic DNA, 10 μl of 2 × SYBR Green qPCR Master Mix (Thermo Scientific), and 0.5 μM primer. Reaction was performed in CFX96 Touch™ Real-Time PCR Detection System (BIORAD), with an initial denaturation at 95 °C for 3 min, followed by 45 cycles at 94 °C for 30 s, 55 °C for 30 s, and 68 °C for 1 min with a final 95 °C for 10 s. This was then followed by a melting curve step of temperature ranging from 65 °C to 95 °C with 0.5 °C increments to determine the melting temperature of each amplified product. Each assay included positive controls of both *P. falciparum*7G8 (MRA-926) and HB3 (MRA-155) isolates as well as *P. vivax* Pakchong (MRA-342G) and Nicaragua (MRA-340 g) isolates, in addition to negative controls including uninfected samples and water. A standard curve was produced from 10-fold dilution series of the control plasmids (*P. falciparum and P. vivax*) and laboratory culture (*P. falciparum)* ranging from 1 % to 1.75 × 10^−12^% to evaluate qPCR efficiency as well as to extrapolate parasite density from gene copies. Melting curve analyses were performed for each amplified sample to confirm specific amplifications of the target sequence. The slope of the linear regression of threshold cycle number (*C*_*t*_) versus log_10_ (Gene Copy Number) was used to calculate amplification efficiency (E). The amplification efficiency ranges from 92 ± 2 % among all runs. For the measure of reproducibility of the threshold cycle number (*C*_*t*_), the mean *C*_*t*_ value was calculated from triplicates in two independent assays. A cutoff threshold of 0.02 fluorescence units that robustly represented the threshold cycle at the log-linear phase of the amplification and above the background noise was set to determine *C*_*t*_ value for each sample. Samples yielding *C*_*t*_ values higher than 40 (as indicated in the negative controls) were considered negative for *Plasmodium* species. The parasite gene copy number (GCN) in a sample was quantified based on the threshold cycle using the follow equation: $\mathsf{G}\mathsf{C}{\mathsf{N}}_{\mathsf{sample}} = {{\mathsf{e}}^{\Big[}}^{\mathsf{E}\times \varDelta \mathit{\mathsf{C}}\mathit{\mathsf{t}}\mathsf{sample}\Big]}$; where GCN stands for gene copy number, Δ*C*_*t*_ for the difference in threshold cycle between the negative control and the sample, and E for amplification efficiency.

### Microsatellite genotyping

For patients who showed residual or recurred parasitemia after treatment, multilocus genotypes based on microsatellites were compared between samples collected at day 0 (before treatment) and the day that indicated recurred parasitemia subsequent to treatment. Thirteen single-copy microsatellites with tri- or tetranucleotide repeats, which mapped to 14 chromosomes,were typed for *P. falciparum*. Alleles were PCR-amplified with the published oligonucleotide primers [44, 45]. For each PCR reaction, 2 μl of genomic DNA were used with 2 mM MgCl_2_, 2 μM of each primer, 0.1 mM of each dNTP, 1 U of recombinant Taq polymerase, and 10 μl of 2 × Taq polymerase buffer in a final volume of 20 μl. All reagents were purchased from Thermo-Scientific, except for primers (both labeledwith fluorescent dyes and unlabeled), which were supplied by Applied Biosystems (Foster City, CA). PCR cycling conditions were as follow: 2 min, 94 °C; (30 s, 94 °C; 40 s, 58 °C; 50 s, 72 °C) for 40 cycles; 5 min, 72 °C. After PCR amplification, products were pooled as follows: TAA87 + PFPK2 + POLY2 + 9735, TA1 + TAA42 + TA81 + TA109, PE87a + PfG377 POLYα + TA124, TA80 + TA116 according to their sizes and fluorescent labels. All alleles were determined and visualized in Peak Scanner. The identity or differences in genotypes allowed us to determine whether recurred parasitemia was attributed to the same or new parasite strain(s) after treatment.

### Resistance gene sequencing of *P. falciparum*

To examine the association between resistance gene mutations and parasitemia clearance time, five gene regions (*pfcrt*, *pfmdr*1, *pfatp*6, and *pfmrp*1, and *pf*K13) that are putatively associated with ACT resistance, were sequenced with *P. falciparum* day 0 samples (before anti-malarial treatment). Polymorphisms were examined for the following codons of each respective gene: *pfcrt* gene –codon76; *pfmdr1*– codons 86, 184, 1034, 1042, and 1246; *pfatp6*– codons 37, 89, 693, 769; *pfmrp1*– codons 191, 437,866, 876, 1390 and 1466; *pf*K13 – codon446 (of which mutant was shown to be prevalent in Myanmar) [31, 32]. Amplification was conducted in a 20 μl reaction mixture containing 3 μl of genomic DNA, 12.5 μl of 2 × DreamTaq Green PCR Master Mix (Thermo Scientific, Waltham, MA), and 10 nmol of forward and reverse primes. We used the primers as well as the PCR conditions of the published protocols [31, 46–50]. PCR products were then purified the by the SAP-ExoI method (Affymetrix, Santa Clara, CA) and sequenced in both directions by Sanger sequencing (GENEWIZ).

### *Pfmdr*1 gene copy estimation

The *pfmdr*1 gene copy number of *P. falciparum* day 0 samples were assessed by real-time PCR. Genomic DNA of *P. falciparum* clones 3D7 (which has a single copy of *pfmdr*1) was used as a calibrator and *pfβ-tubulin*, a house-keeping gene, was used as an internal control. The primers for the amplifications of*pfmdr*1 and *β-tubulin* were described previously [51]. Amplification was performed in triplicate in a total volume of 20 μl containing 10μlof SYBR Green PCR Master Mix, 0.75 μl of each of the sense and anti-sense primers (10 μM), 20 ng of genomic DNA and 3.5 μl of water. PCR condition was as follow: 95 °C for 10 min, followed by 40 cycles at 95 °C for 15 s and at 60 °C for1 min. A negative control with no template was used in each run. Each sample was run in triplicates and the *C*_*t*_ values and melting temperature were recorded at the end of the reactions. The average and standard deviation of the three *C*_*t*_ values were calculated, and the average value was accepted if the SD was lower than 0.32. In this study, the 2^-ΔΔCt^ method for relative quantification [52] was used to estimate the copy numbers of *pfmdr*1 gene by the following equation: ${}^{\boldsymbol{\Delta} \boldsymbol{\Delta}}{C}_t = {\left({C_t}_{\mathsf{target}\ \mathsf{gene}}\hbox{--} {C_t}_{\mathit{\mathsf{p}}\mathit{\mathsf{f}}\mathit{\mathsf{b}}\mathit{\hbox{-}}\mathit{\mathsf{tubulin}}}\right)}_{\mathsf{unknown}\ \mathsf{sample}}\hbox{--}\ {\left({C_t}_{\mathsf{target}\ \mathsf{gene}}\hbox{--} {C_t}_{\mathit{\mathsf{p}}\mathit{\mathsf{f}}\mathit{\mathsf{b}}\mathit{\hbox{-}}\mathit{\mathsf{tubulin}}}\right)}_{3\mathrm{D}7}$. The result for each sample was expressed in N-fold changes in unknown samples (2^-ΔΔCt^). A minimum of two independent runs was conducted for each sample and the results were expressed as the N-fold copy number of a given gene relative to *P. falciparum* 3D7 by calculating the mean between the two runs. N-fold copy numberbetween0.8 and 1.4 was considered as a single copy and N-fold copy number greater than 1.5 was considered as multiple copies of the target gene [52, 53].

### Statistical analyses

Ordered logistic regression, both univariate and multiple, was used to analyze the association between clearance time and resistance gene mutations. The following combination of orders of parasite clearance time were tested: i) parasite cleared within 3 days (order 1), 7 days (order 2), 14 days (order 3), not cleared (order 4); ii) parasite cleared within 3 days (order 1), 7–14 days (order 2), not cleared (order 3); iii) parasite cleared within 3 days (order 1), 7 days (order 2), 14 days or not cleared (order 3); iv) parasite cleared within 3 days (order 1) and the rest (order 2). In addition, Fisher’s exact test (given small sample size) was used to test for significant differences in age (below and above 18) and initial parasitemia between samples that showed fast and delayed parasite clearance time. All statistical analyses were performed in R (R Core Team 2013).

## Results

### Change in parasitemia after initial drug treatment

**Fig. 1:**
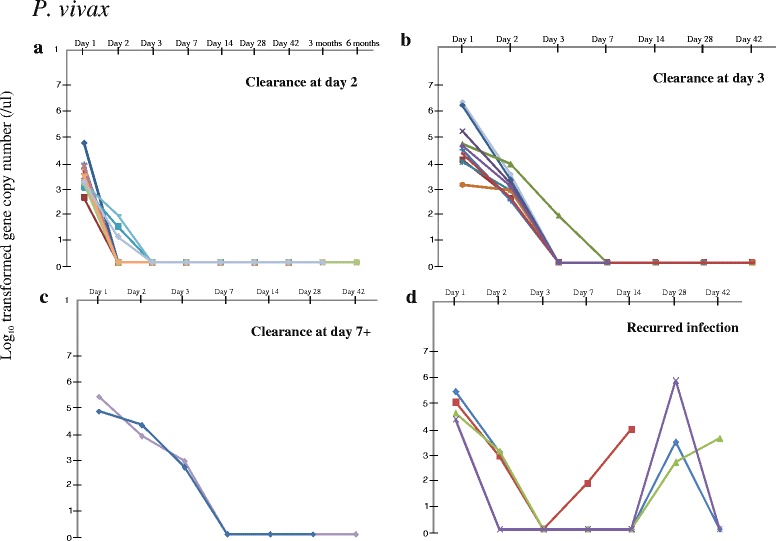
Change in *Plasmodium vivax* parasitemia among follow-up samples collected from day-0 (the day when the patient was admitted to the hospital and sought antimalarial treatment) to up to 6-months after treatment. Samples with cleared parasitemia at day-2 **a**, day-3 **b**, at day-7 and after **c**, and with recurred parasitemia **d** were presented. For the samples that showed recurred infection, the initial and recurred genotypes were found to be different based on microsatellites, suggestive of a newly infected parasite strain that caused recurred parasitemia after initial drug treatment

For the 130 patients who were diagnosed with *P. vivax*, 124 (95.4 %) showed cleared parasitemia at days 2 or 3. These samples indicated a PRR_48_ value of <0.01, which means over 99 % of the initial parasitemia was cleared after 48 h of treatment. Only two samples (1.5 %) showed delayed clearance where residual parasitemia remained at day 3 but cleared at day 7 (Table 1; Fig. 1). Four of the samples indicated initial decline but recurred parasitemia at day 14 and 28 (Fig. 1). These four samples showed different microsatellite genotypes between the initial and recurred infections, suggestive of a newly infected parasite strain that caused recurred parasitemia after initial drug treatment.

**Table 1:** Parasite clearance and recurred infection of *P. falciparum* and *P. vivax* cases in Myanmar

*P. falciparum*			
IDP settlement			
	Clearance time		No. of cases (%)
		2 days	11 (29.7)
		3 days	12 (32.4)
		7+ days	12 (32.4)
	Recurred infection		2 (5.4)
		Total	37
Village			
	Clearance time		No. of cases
		2 days	9 (34.6)
		3 days	5 (19.2)
		7+ days	8 (30.7)
	Recurred infection		4 (15.4)
		Total	26
*P. vivax*			
IDP settlement			
	Clearance time		No. of cases
		2 days	78 (74.3)
		3 days	21 (20)
		7+ days	2 (1.9)
	Recurred infection		4 (3.8)
		Total	105
Village			
	Clearance time		No. of cases
		2 days	25 (100)
		3 days	0
		7+ days	0
	Recurred infection		0
		Total	25

**Fig. 2:**
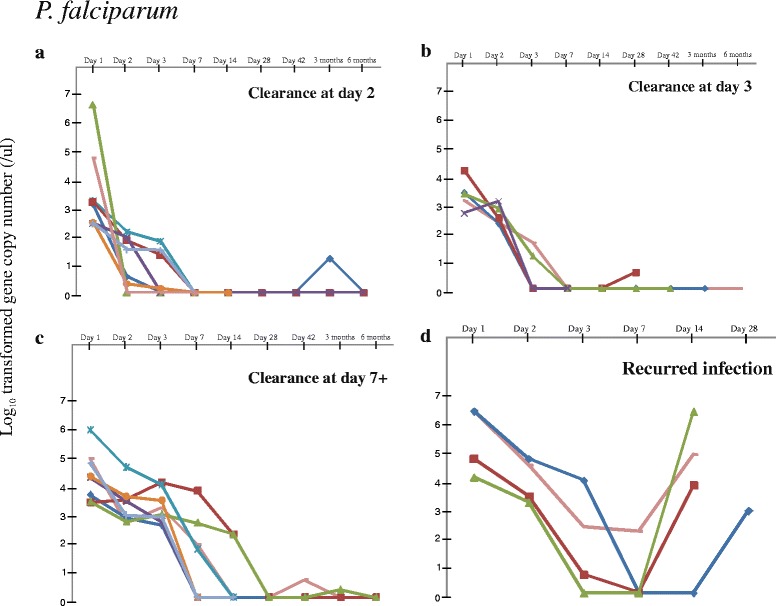
Change in *Plasmodium falciparum* parasitemia among follow-up samples collected from day-0 (the day when the patient was admitted to the hospital and sought antimalarial treatment) to up to 6-months after treatment. Samples with cleared parasitemia at day-2 **a**, day-3 **b**, at day-7 and after **c**, and with recurred parasitemia **d** were presented. For the samples that showed recurred infection, two samples showed identical microsatellite genotypes

For the 63 patients diagnosed with *P. falciparum*, 37 (58.7 %) showed cleared parasitemia at day 2 or 3 after treatment (Table 1; Fig. 2); 20 (31.7 %) showed delayed clearance where residual parasitemia was detected at day 3 but cleared at day 7; and six (9.5 %) showed initial decline but recurred parasitemia after day 14 of the treatment (Fig. 2). Results based on PRR_48_ were largely consistent with those based on day-3 positivity. All samples that were defined as fast clearance (cleared on day 2 or 3) indicated >99 % parasite clearance after 48 h. For samples that showed delayed clearance, parasites were only reduced to >99 % after day-3 with the exception of one sample that showed >99 % clearance at day-3 after normalization with initial parasitemia.

**Fig. 3:**
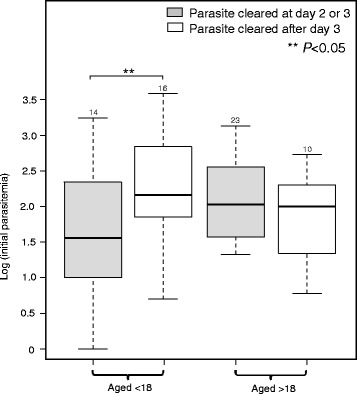
Boxplot comparing initial parasitemia of *P. falciparum* samples that indicated fast (gray box; parasite cleared at day 2 or 3) and delayed (white box; parasite cleared after day 3) parasite clearance between two age groups (aged below and under 18). Number above bar indicates number of samples included. Asterisk indicates level of significance

When we stratified our samples by age, a greater proportion of samples that displayed fast parasite clearance time were adults (23 out of 37; Additional file 1), whereas a greater proportion of samples that displayed delayed parasite clearance time belong to the younger age group (16 out of 26). Such differences, however, were not significant likely due to small sample size. Interestingly, samples from the younger age group with delayed clearance time indicated a significantly higher initial parasitemia compared to those with fast clearance time (Fig. 3). Nonetheless, the level of initial parasitemia did not shown to be significantly different by parasite clearance time in adults.

**Fig. 4:**
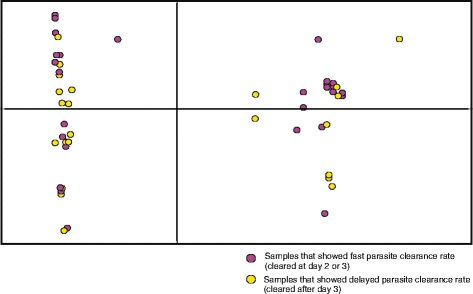
Scatter plot based on Principal Component Analysis (PCA) of microsatellite data among the fast (purple color; no parasite detected at day-3) and delayed (yellow color; parasite detected after day-3) clearance samples of *P. falciparum*

Among the recurred infections, four showed microsatellite genotypes different from the initial infections, suggestive of a newly infected parasite strain that caused recurred parasitemia; whereas the remaining two samples showed identical genotypes. Between samples that showed fast (parasite cleared at day 2 or 3) and delayed (parasite observed at day3 or after) clearance of *P. falciparum*, no genetic differentiation was observed based on microsatellite loci despite that these samples were clustered by geographical sites (Fig. 4).

### Resistance gene polymorphisms in *P. falciparum*

**Fig. 5:**
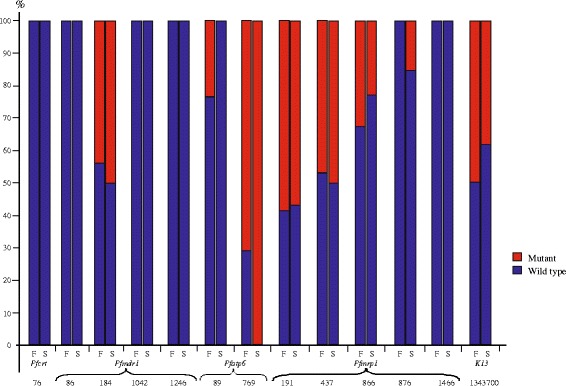
Frequency of mutations in various gene codons among samples that showed fast (parasite cleared at day 2 or 3) and delayed (parasite cleared after day 3) clearance of *P. falciparum*. Bold denotes codon of which the mutation frequency is significantly associated with the parasite clearance time

Because only two out of the 130 *P. vivax* samples indicated delayed parasitemia clearance, resistance gene mutation was not examined on *P. vivax*. For the 63 *P. falciparum* samples that indicated varied parasite clearance time, all had the wild type genotype K76 of the *pfcrt* gene (Fig. 4; Additional file 2). Likewise, for *pfmdr*1all samples showed the wild type N86, N1042, and D1246, except for codon 184, of which approximately 50 % of the patients with fast and delayed parasite clearance showed Y184 and 184 F, respectively (Fig. 5; Additional file 2). Nevertheless, based on regression analyses, mutation at this codon position was not significantly associated with delayed clearance in our samples (Additional file 3). Our qPCR data indicated that patients with delayed parasite clearance contained almost an equal proportion of single, duplicate, and more copies of the *pfmdr*1 gene, whereas those with fast parasite clearance contained mostly two or more copies. The difference observed in *pfmdr*1 copy number was not shown to be significantly associated with parasite clearance time.

Amplification and sequencing of the entire *pfatp*6 gene indicated polymorphisms at codons 89 and 769, but no mutations at codons 37, 639, and 898 among the *P. falciparum* samples. For codon 89, the majority of the samples (86.7 %) showed to have the wild typeI89 genotype, while the remaining eight samples (fast clearance) had the mutant 89 T (Fig. 5; Additional file 2). On the other hand, for codon 769,70.6 % of the patients with fast parasite clearance and all of those with delayed clearance had the mutant 769A, whereas only 10 samples (patients with fast clearance) had the wild type D769 genotype (Fig. 5; Additional file 2). These mutations were shown to be not significantly associated with difference in the parasite clearance time.

For the *pfmrp*1 gene, polymorphisms were detected at four codon positions (H191Y, S437A, H866N, and I876V). The proportion of wild type and mutant at codons 191 and 437 were roughly 50 %, respectively, among the patients with fast and delayed parasite clearance, whereas at codons 866 and 876 over 65 % and 80 % of the samples showed to have the wild type respectively (Fig. 5; Additional file 2). No samples indicated any mutation at codon 1466 but wild type K1466, similar to the findings of Pirahmadi et al. [50]. Among all the detected polymorphisms, logistic regression analyses indicated significant association between the mutation in codon 876 of *Pfmrp*1 and clearance time (*χ*^2^ = 7.92, d.f. = 1, *P* = 0.005).

For the small fragment of the *K13* gene, mutations were detected at the amino acid position 446, of which 66 % had the wild type F and 34 % had the mutant I among all samples. However, this mutation was not significantly associated with the parasite clearance time.

## Discussion

In the present study, blood samples of malaria patients who received antimalarial treatment were monitored for at least 28 days. The efficacy of antimalarial therapy was interpreted by the reduction of the parasite load for these patients. Changing patterns of morphological appearances of parasite species possibly due to drug pressure or strain variation can present difficulty to quantify parasite load by microscopy [54]. Quantifying parasitemia based on qPCR provides a sensitive means in measuring samples of low or submicroscopic parasitemia particularly during antimalarial treatment [43]. However, it is noteworthy that in the present study human DNA was not amplified as internal controls for our samples. Despite that amplification was done in triplicate for each of our samples, we cannot rule out the possibility that DNA extraction or PCR errors may slightly influence the quantification of parasite DNA.

In Southeast Asia, artemisinin derivatives have been used for more than two decades as first-line malaria treatment. However, recent reports of delayed parasite clearance after artemisinin-based treatment raises concerns about the effectiveness of the drug as well as the spread of resistance especially in malaria endemic countries [8, 55, 56]. For instances, previous studies have shown delayed parasite clearance after ACT treatments in southeastern Myanmar and border area of Myanmar-Thailand where artemisinin has been used for several years [11, 32, 57–60]. A study by Wang et al. [61] demonstrated an overall 42-day cure rate of 100 % for DP treatment of uncomplicated *P. falciparum* malaria at the China-Myanmar border area and a day-3 parasite-positive rate of 7 %. Up to 18 % (13/71) of the patients showed detectable gametocytes and a large proportion of them were persistent from the first 3 days of antimalarial treatment [61]. In Thailand, parasites with delayed clearance after ACT did not show increased resistance to artemisinin compounds based on conventional in vitro experiments [8]. In Western Cambodia, there is evidence that *P. falciparum* parasites clear slowly from the blood after ACT treatment and that the variation in clearance rate is largely explained by genotypic differences observed among parasite strains [55], despite factors such as host immunity and splenic function that cannot be ruled out. Microsatellites indicated that our *P. falciparum* samples were genotypically differentiated by sites specifically between the IDP settlement and local village in Myanmar but not differentiated by parasite clearance time of the samples. Age has been shown as a factor influencing parasite clearance time [37, 62]. It is possible that higher initial parasitemia associated with multiple infections and/or weaker immunity in the younger age group could delay the parasite clearance time. This observation merits further investigation with expanded samples.

The development of resistance to antimalarial drug in a parasite is a multifactorial molecular process and more than a single gene could be involved in reduced susceptibility. Various mutations in genes such as *pfatp*6, *pfmdr*1 and *pfmrp*1 have been suggested to account for ACT resistance. Recently, mutations in the *Kelch*-13 propeller gene were proposed to be involved in ACT resistance. In Mynamar, ACT has been used as first-line antimalarial treatment since the 1990s subsequent to decline in clinical efficacy of CQ and SP [3]. Earlier studies showed that complete withdrawal or reduced usage of CQ as first-line antimalarials can result in a decreased prevalence of *pfmdr*1 86Y and *pfcrt* 76 T mutations [63, 64]. Despite our small sample size, our data agrees with this finding and reveals a dominance of wild type genotypes in both genes (except *pfmdr*1 codon 184) that relate to CQ resistance among the *P. Falciparum* samples. Given that CQ has not been used for more than a decade in Mynamar, a relaxation of selective pressure likely resulted in high susceptibility of *P. falciparum* to this drug.

The role of *pfmdr*1 gene mutations in artemisinin-based drug resistance is unclear. Previous in vivo studies showed that mutations at codons 86 and 1246 play an important role in the resistance of *P. falciparum* to mefloquine and artemisinin [65]. In the Thai-Myanmar border region, 1226Y mutant was prevalent among *P. falciparum* parasites and significantly associated with in vitro response to artemisinin [30]. However, these mutations were not observed in our samples. The 184 F allele was reported to be associated with increased IC_50_ of artesunate based on in vitro study [66]. Approximately 86 % of the 184 F allele was reported in western Cambodia where the level of MQ resistance was significant [67]. Imwong et al. [68] has also shown that 184 F of *pfmdr*1 is the only mutation associated with slow parasite clearance rates, despite the fact that such association did not persist when the results were adjusted by site. Although184F was found to be prevalent among our samples, this mutation was not significantly correlated with the parasite clearance time in the present study. Furthermore, while various studies showed that increased *pfmdr*1 gene copy number is significantly related to a reduced sensitivity of *P. falciparum* to mefloquine, quinine, and artesunate resistance [18, 30, 53, 69], our samples showed no significant correlation between gene copy number and parasite clearance time.

Previous studies suggested that mutations in the gene *pfatp*6, which encodes the sarcoplasmic and endoplasmic reticulum Ca^2+^ -ATPase (SERCA)-type protein in *P. falciparum* may alter the parasite sensitivity to artemisinin [70]. For instances, Jambou et al. [27] reported a significant decrease in *invitro* sensitivity to artemether in *P. falciparum* isolates from French Guiana and that this reduced efficacy was associated with a S769N polymorphism in the *pfatp*6 gene. Several polymorphisms have also been identified in the *pfatp*6 gene including the mutations E431K and A623E in Senegal [71], I89T in Thailand [53], H243Y in Central Africa [72], T2694 in São Tomé and Principe [52], as well as R37K and A630S in Brazilian Amazon [73]. In 2008, Dahlstrom et al. [28] identified 33 single nucleotide polymorphisms (SNPs), three of which were found in a frequency higher than 5 % in codons H431K, N569K and A630S among the *P. falciparum* isolates from East and West Africa. In this study, polymorphism was detected only in codons 89 and 769. However, no significant correlation was observed between these mutations and parasite clearance time. While our small sample size may have hidden other possible mutations or underestimated the frequency of the observed mutations, it is also possible that *pfatp*6 does not play a key role in ACT resistance as shown in recent studies [29, 73, 74].

Apart from the *pfatp*6, *pfmrp1* from the ATP-binding cassette (ABC) family of transporters has recently emerged as a potential genetic target for multiple drugs. The mutations in the gene *pfmrp1*have been shown to be associated with resistance to chloroquine, quinine, sulfadoxine/pyrimethamine and artemisinin derivatives in *P. falciparum* [24, 25, 66, 75–78]*.* Although recent studies indicated that mutations 1390I and 1466 K were associated, respectively, with artemisinin and SP resistance [78], our samples all showed wide type allele in these codon positions despite the limited sample size. These results suggest either a marked reduction of selection pressure with these antimalarials in the study area or there are other mutations that play a more vital role in determining resistance. In this study, the only mutation that was significantly associated with delayed parasite clearance is 876 V. Although this mutation has been shown to play a significant role in changing the functionality of the protein [78] and recent studies have reported its association with in vivo ACT response [24, 30] as well as in vitro susceptibility to chloroquine [34], the low frequency of this mutation among our samples suggests potentially other mutations that were not examined here are responsible for delayed clearance.

A recent population study of the *K*13-propeller polymorphisms has shown a predominant F446I mutation in *P. falciparum* from the China-Myanmar border area [31, 32]. This mutation was shown to be equally prevalent in our patients with fast and delayed parasite clearance, and no association was found between its mutation and delayed parasite clearance time. The full sequence of the *K13* gene will be examined on broad samples based on pyrosequencing and the frequency of other mutations will be reported elsewhere.

## Conclusions

The majority of the *P. vivax* infections showed parasite clearance at day-2 or −3 subsequent to first day drug treatment, indicative of continual effectiveness of chloroquine on *P. vivax* in Myanmar. By contrast, over 40 % of the *P. falciparum* infections indicated parasite positivity after ACT drug treatment. This raises concern to the present antimalarial treatment of *P. falciparum* malaria in combat with the emergence and spread of ACT resistance.

### Ethics statement

Scientific and ethical clearance was given by the institutional scientific and ethical review boards of Kunming Medical University, China; University of California at Irvine, USA; Pennsylvania State University, USA; and the Bureau of Health of Kachin State, Myanmar. Written informed consent/assent for study participation was obtained from all consenting heads of households or parents/guardians (for minors under age 18) and from each individual who was willing to participate in the study.

### Availability of data and materials

The age distribution of patients that showed fast as well as delayed parasite clearance can be found in Additional file 1. Mutation type of the different codon positions of each sample can be found in Additional file 2. The frequency of mutations in various gene codons among the study samples can be found in Additional file 3.

## Additional files

Additional file 1: Histogram showing the number of patients that indicated fast parasite clearance (cleared at day 2 or 3) as well as delayed clearance (parasite cleared after day 3) with respect to two age groups (blue: aged below 18; red: aged above 18). The level of significance was indicated. (EPS 280 kb) Additional file 2: Mutation type of each of the targeted codon positions of respective genes as well as *mdr*1 gene copy number among fast and delay clearance *P. falciparum* samples. (XLSX 15 kb) Additional file 3: Frequency of mutations in various gene codons among samples that showed fast (parasite cleared at day 2 or 3) and delayed (parasite cleared after day 3) clearance of *P. falciparum*. Bold denotes codon of which the mutation frequency is significantly associated with the parasite clearance time. (DOCX 77 kb)

## Abbreviations

ACT: Artemisinin Combined Therapy
atp: adenosine triphosphate
CQ: chloroquine
crt: chloroquine resistance transporter
dhfr: dihydrofolate reductase
dhps: dihydropteroate synthase
DP: dihydroartemisinin-piperaquine
GCN: gene copy number
GMS: Greater Mekong Subregion
mdr1: multi-drug resistance gene
mrp: multidrug-resistance protein
MQ: mefloquine
PCT: Parasite Clearance Time
PRR: parasite reduction ratio
qPCR: quantitative Real-Time PCR
SP: sulphadoxine and pyrimethamine
