# Supplementary material for: Examining Plasmodium falciparum and P. vivax clearance subsequent to antimalarial drug treatment in the Myanmar-China border area based on quantitative real-time polymerase chain reaction
Source: BMC Infect Dis. 2016 Apr 16;16:154. doi: 10.1186/s12879-016-1482-6 (PMC4833920; doi:10.1186/s12879-016-1482-6)
Supplement: Additional file 3: — Frequency of mutations in various gene codons among samples that showed fast (parasite cleared at day 2 or 3) and delayed (parasite cleared after day 3) clearance of P. falciparum. Bold denotes codon of which the mutation frequency is significantly associated with the parasite clearance time. (DOCX 77 kb) [file 12879_2016_1482_MOESM3_ESM.docx]

Supplementary Table 1. Frequency of mutations in various gene codons among samples that showed fast (parasite cleared at day 2 or 3) and delayed (parasite cleared after day 3) clearance of *P. falciparum*. Bold denotes codon of which the mutation frequency is significantly associated with the parasite clearance time

| Gene | Codon | Frequency (%) | |
| --- | --- | --- | --- |
|  |  | Fast clearance (*n*=34) | Slow clearance (*n*=26) |
| *Pfcrt* |  |  |  |
|  | K76T | K: 34; T: 0 | K: 26; T: 0 |
| *Pfmdr1* |  |  |  |
|  | N86Y | N: 34; Y:0 | N: 26; Y:0 |
|  | Y184F | Y: 19; F: 15 | Y: 13; F: 13 |
|  | 1034 |  |  |
|  | N1042D | N: 34; D: 0 | N: 26; D: 0 |
|  | D1246Y | D: 34 Y: 0 | D: 26; Y: 0 |
| *Pfatp6* |  |  |  |
|  | R37K | R: 34; K: 0 | R: 26; K: 0 |
|  | I89T | I: 26; T: 8 | I: 26; T: 0 |
|  | A630S | A: 34; S: 0 | A: 26; S: 0 |
|  | D769A | D: 10; A: 24 | D: 0; A: 26 |
| *Pfmrp1* |  |  |  |
|  | H191Y | H: 14; Y: 20 | H: 11; Y: 15 |
|  | S437A | S: 18; A: 16 | S: 13; A: 13 |
|  | H866N | H: 23; N: 11 | H: 20; N: 6 |
|  | **I876V** | **I: 34; V: 0** | **I: 22; V: 4** |
|  | F1390I | F: 34; I:0 | F: 26; I:0 |
|  | K1466R | K: 34; R: 0 | K: 26; R: 0 |
| *K13* |  |  |  |
|  | F446I | F: 17; I: 17 | F: 16; I: 10 |
